# Infliximab-Induced Interstitial Pneumonitis: A Case Report

**DOI:** 10.7759/cureus.40812

**Published:** 2023-06-22

**Authors:** Eyad A Makkawy, Manal T Alanazi, Mohamed F Yehia, Abdullah M Almazloum

**Affiliations:** 1 Internal Medicine/Gastroenterology, Prince Mohammed Bin Abdulaziz Hospital, Riyadh, SAU; 2 Gastroenterology, Prince Mohammed Bin Abdulaziz Hospital, Riyadh, SAU; 3 Pulmonology, Prince Mohammed Bin Abdulaziz Hospital, Riyadh, SAU

**Keywords:** anti-inflammatory therapies, anti-tumor necrosis factor, pneumonitis, interstitial pneumonitis, infliximab

## Abstract

Anti-tumor necrosis factor inhibitors are increasingly being recommended to treat and control a wide range of diseases, including Crohn's disease, ulcerative colitis, rheumatoid, and psoriatic arthritis. Serious pulmonary consequences, ranging from infectious disease to pulmonary edema, airway involvement, and even interstitial lung disease, are well-known multisystemic side effects. Interstitial lung disease is a well-known but uncommon condition.

This report presents a case of a 49-year-old man with ulcerative colitis who developed interstitial pneumonitis following three infusions of infliximab therapy based on clinical, radiologic, and pathology data that are consistent with drug-induced interstitial pneumonitis. After stopping infliximab and starting steroid therapy, we noticed complete symptom resolution and improvement in respiratory symptoms and imaging.

## Introduction

Inflammatory bowel diseases (IBDs) are now typically treated with anti-inflammatory treatments, including biologics. The Food and Drug Administration (FDA) has authorized infliximab (Remicade, Inflectra) for use in children aged six years and older [1].

The increasing use of anti-tumor necrosis factor-alpha (TNF-alpha) agents in the last decades is associated with known side effects, some of them related to the development of autoimmune diseases [2]. With this use and longer follow-up periods of treatment, there are a growing number of reports of interstitial lung disease (ILD) related to this therapy [3]. Their consistent use in treating conditions like IBD translates into a new clinical challenge since pulmonary involvement in these patients is not unusual and there is a potential secondary effect of medications employed to control inflammation [4,5].

In the following case report, we give an example of a patient with ulcerative colitis who developed interstitial pneumonitis after receiving infliximab therapy.

## Case presentation

A 49-year-old male presented with a previous medical history of bronchial asthma and extensive ulcerative colitis (that was diagnosed in October 2019), five months prior to his admission to our hospital. He had received mesalamine oral 3 gm daily, mesalamine rectal suppository 1 gm daily at bedtime, and prednisolone 40 mg once daily with a tapering dose of 5 mg every week for two months, without having any pulmonary manifestations.

In outpatient follow-up, the patient improved minimally and a sigmoidoscopy was done for re-evaluation. The patient still had active disease based on clinical, endoscopic, and histopathologic findings. After that, because of inadequate control of the disease, the patient was started on biological therapy (infliximab). He received three loading doses at intervals of zero, two, and six weeks.

**Figure 1:**
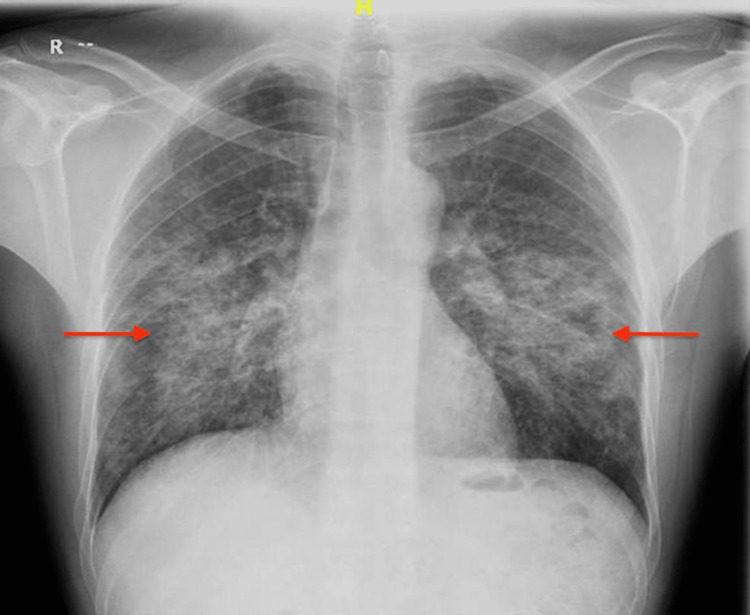
Chest X-ray Bilateral heterogeneous opacities and clear costophrenic angles.

He visited the emergency department (ED) one month after receiving his third infliximab dose with the major symptom of progressively worsening shortness of breath. He denied having orthopnea, swollen legs, weight increase, fever, appetite loss, night sweats, palpitations, recent travels, or encounters with sick people. He was found to be feverish and tachypneic, and 2 liters of oxygen was administered through a nasal cannula to keep his saturation level at 97%. No jugular venous distention or wheezes were seen. He was crepitating on both sides. The initial chest X-ray revealed costophrenic angles and bilateral heterogeneous opacities that were not seen on earlier radiographs (Figure 1).

**Figure 2:**
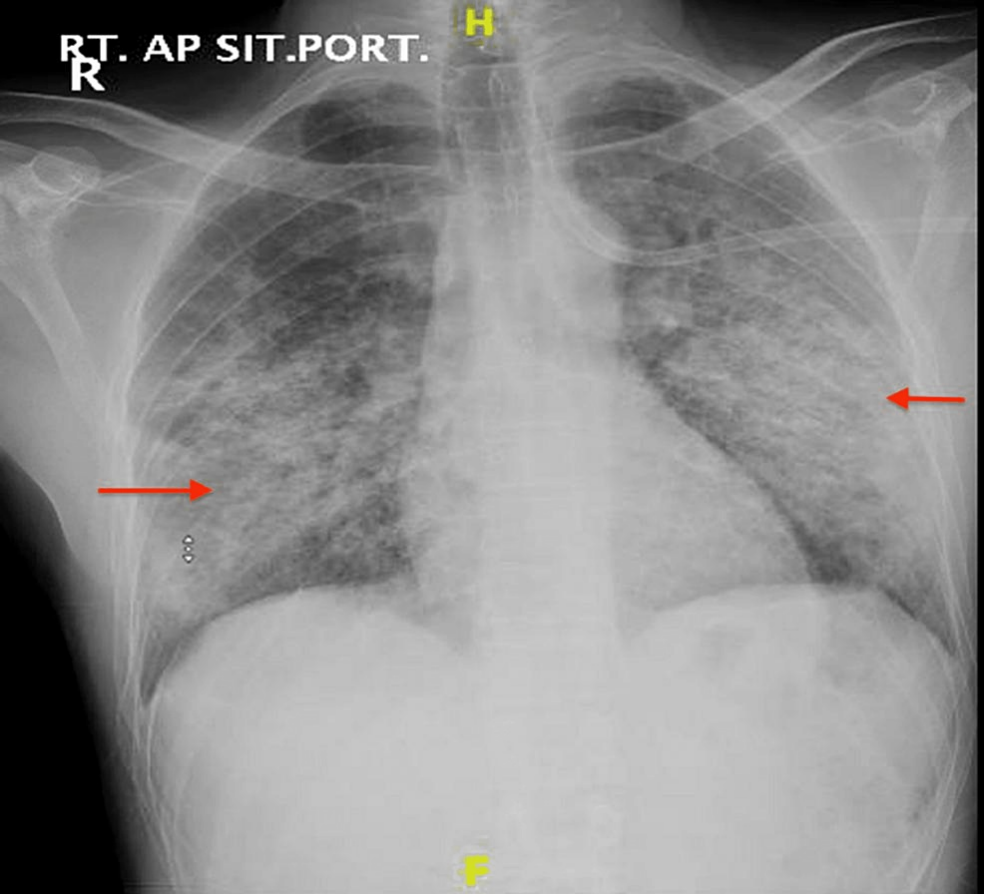
Chest X-ray obtained in the ICU

**Figure 3:**
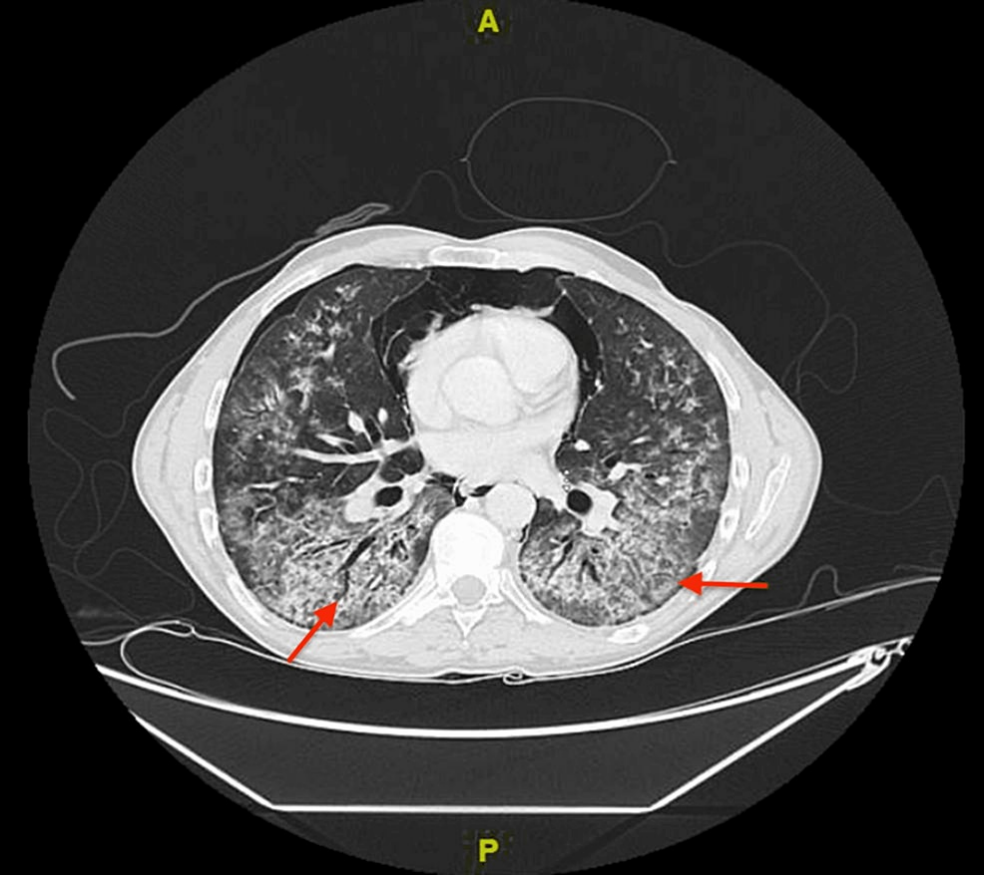
Chest CT scan Chest CT with contrast showed patchy bilateral dependent ground glass opacities with associated tree-in-bud nodules along with interlobular septal thickening and traction bronchiectasis suggestive of lung inflammation on top of interstitial lung disease.

**Figure 4:**
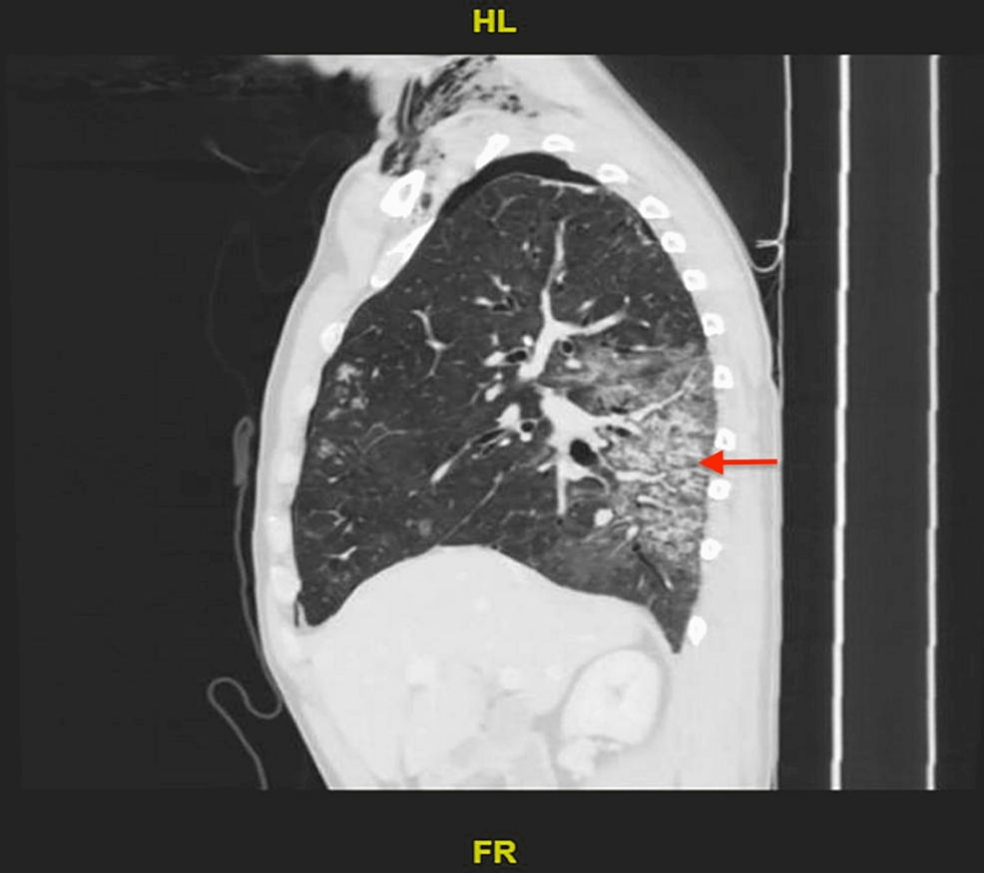
Chest CT scan

**Figure 5:**
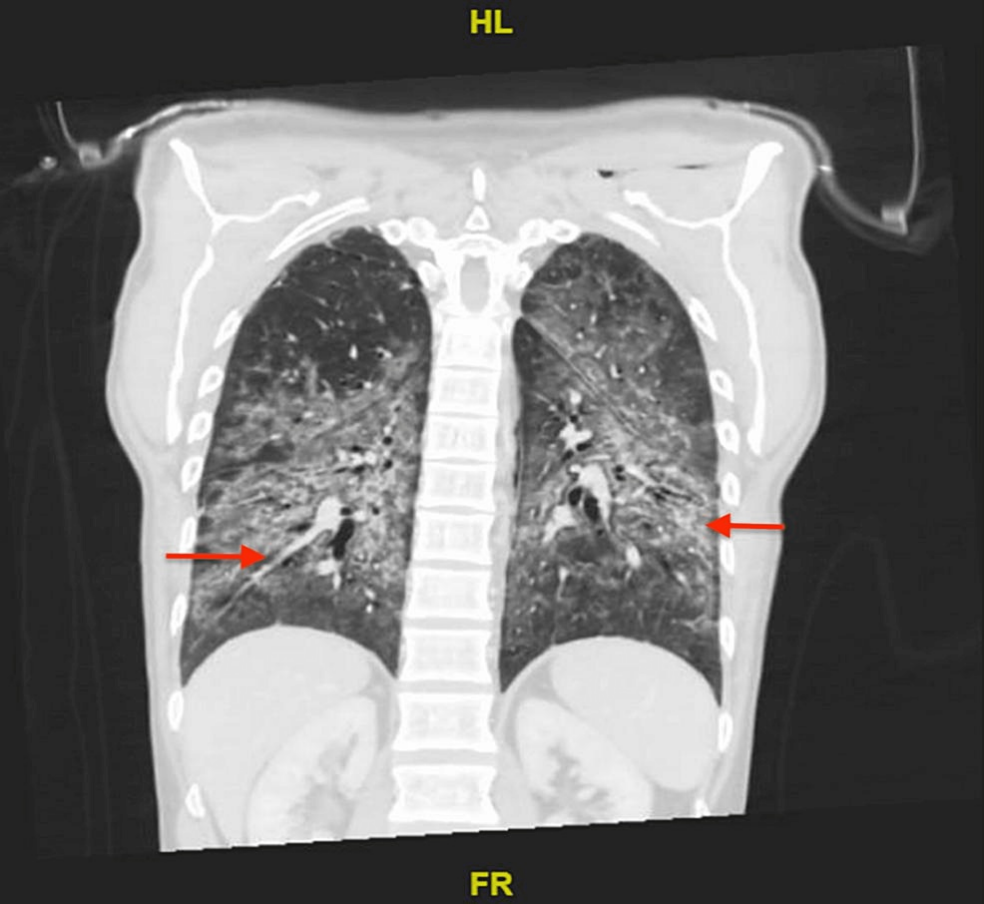
Chest CT with contrast Bilateral bronchiectasis affecting the lower lobes. There are patchy bilateral dependent ground glass opacities with associated tree-in-bud nodules along with interlobular septal thickening and traction bronchiectasis.

The patient was admitted for further evaluation. He was treated with budesonide/formoterol and salbutamol nebulizers. Ceftriaxone and azithromycin were started for empiric antibiotic coverage for community-acquired pneumonia. His symptoms did not resolve with supplemental oxygen and nebulizer treatment. The patient was desaturated and had an increased requirement of oxygen at 10 liters by facemask. Chest X-ray showed worsening in bilateral heterogeneous opacities (Figure 2) and chest CT with contrast showed patchy bilateral dependent ground glass opacities with associated tree-in-bud nodules along with interlobular septal thickening and traction bronchiectasis suggestive of lung inflammation on top of ILD (Figures 3-5). Despite upgrading antibiotics to trimethoprim/sulfamethazine and piperacillin/tazobactam, the patient’s symptoms did not resolve and a bronchoscopy was performed. Bronchoalveolar lavage was negative for malignant cells, *Pneumocystis jirovecii* pneumonia (PJP), fungus, and mycobacterium. Transbronchial biopsy was consistent with acute and chronic inflammatory cells.

Based on clinical, radiologic, and pathology data, the patient's condition was consistent with drug-induced interstitial pneumonitis. After nebulizers and antibiotics failed to provide symptom relief, the patient was started on methylprednisolone for a total of 16 days, beginning with 60 mg twice per day (BID) for three days, 1 g daily for two days, followed by a tapering period of 500 mg daily for one day, 250 mg daily for two days, and finally 60 mg BID for seven days. The patient showed a dramatic response in his respiratory symptoms and was discharged from the hospital after one month of admission on a prednisone taper dose with home oxygen therapy.

**Figure 6:**
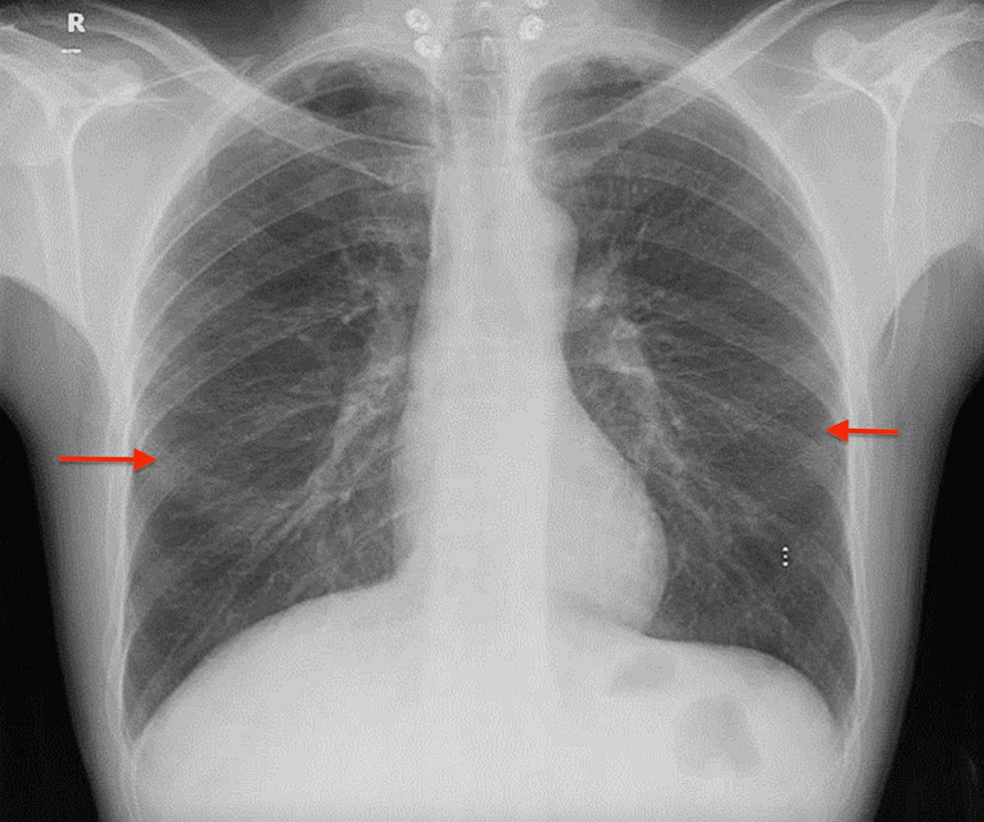
Chest X-ray on outpatient follow-up

One month later in outpatient follow-up, we resumed mesalamine and azathioprine, while his respiratory symptoms were still improving maintaining oxygen saturation on room air. Three months later, the patient was started on vedolizumab for six months but the patient still had inadequate control with a little improvement. So, the patient was shifted to ustekinumab. Currently, the patient is showing a good response to ustekinumab and is in clinical remission (Figure 6).

## Discussion

Through this case, we reported a 49-year-old man with ulcerative colitis who developed interstitial pneumonitis following three infusions of infliximab therapy. To the best of our knowledge, this is the first case reported in Saudi Arabia, in addition to account for most of the cases reported in the literature that showed that the first signs of lung injury appeared between the second and the fourth infliximab infusions [6,7]. In our situation, a substantial temporal correlation between infliximab initiation and rapid onset of ILD implies causality. Despite mesalamine has been linked to lung toxicity [8,9], in our case, the patient has been on mesalamine for a long period, which is unlikely to be the culprit. Our patient was given mesalamine for five months and did not experience any respiratory symptoms during treatment and it was resumed again after the resolution of his pulmonary problem. While after the third dose of infliximab, he began to experience dyspnea when he exerted himself. The reactivation of hidden opportunistic infections, such as tuberculosis and fungal infections, has been the main worry with infliximab usage. These options were not considered in the situation of our patient [10].

Diagnosis of infliximab-induced interstitial pneumonitis was made based on consistent timing of exposure and onset of symptoms, suggestive imaging, and bronchoalveolar and pathological patterns [11,12].

The exact pathogenic processes behind the development or exacerbation of ILD in response to TNF-alpha remain unknown. TNF-alpha may have both profibrotic and antifibrotic actions, according to experimental research. On one hand, it may have antifibrotic effects by reducing pulmonary inflammation as a result of the lung's inflammatory cells dying. The absence of TNF-alpha can result in an accelerated form of bleomycin-induced lung fibrosis that may be reversed by the infusion of TNF-alpha, as Kuroki et al. [13] showed using a TNF-alpha knockout mouse model. As a result, ILD develops when TNF-alpha is blocked because apoptosis is prevented, leaving inflammatory cells to survive in the lung. To prevent fibroblast proliferation, TNF-alpha may potentially interact with interleukin-1 and interferon [14]. The activation of an extracellular regulated kinase-specific pathway in fibroblasts, on the other hand, by TNF-alpha may also have profibrotic effects by increasing the production of transforming growth factor (TGF-1) in the lungs [15]. Additionally, transgenic mice with excessive lung TNF-alpha expression experience chronic lymphocytic alveolitis, the severity of which is correlated with TNF-alpha mRNA levels [16].

Collectively, these results may imply that an imbalance between these two TNF-alpha functions may either cause fibrosis in patients with underlying ILD or, on the other hand, stabilize past ILD in people who are prone to it. However, to test this idea, clinical human studies are required [17].

## Conclusions

In the meanwhile, patients should be warned about the possibility of pulmonary toxicity when starting infliximab medication. As a result, clinical surveillance is indicated for patients receiving this form of treatment who develop respiratory symptoms, particularly during the first four infliximab infusions.
